# A pilot study of cdc6 as a biomarker for circulating tumor cells in patients with lung cancer

**DOI:** 10.1002/jcla.23245

**Published:** 2020-04-06

**Authors:** Cheng An, Guijian Liu, Shi Cheng, Bo Pang, Shipeng Sun, Yaying Zhang, Zhongdai Pan, Xixiong Kang

**Affiliations:** ^1^ Laboratory Diagnosis Center Beijing Tiantan Hospital Capital Medical University Beijing China; ^2^ Department of Clinical Laboratory Guang anmen Hospital China Academy of Chinese medical Science Beijing China

**Keywords:** cdc6, lung cancer, peripheral blood mononuclear cell, proliferating cell

## Abstract

**Background:**

Cell division cycle 6 (cdc6) is a key factor of DNA replication initiation license system and a proto‐oncogene. It has been detected in some tumor tissues to aid cancer diagnosis in many research projects. However, it remains unclear that if cdc6 could be detected in the peripheral blood, just like liquid biopsy, in solid tumor patients. The aim of this study is to investigate the possibility of cdc6 as a biomarker for circulating tumor cells in patients with lung cancer.

**Methods:**

We first detected the expression of cdc6 in peripheral blood mononuclear cells (PBMCs) and tumor cells by in situ hybridization with cdc6 RNA probe. Then, we examined the expression of cdc6 in PBMCs from health individual, mononuclear cells from cord blood, or A549 cell line by RT‐qPCR. Furthermore, we used RT‐qPCR to test the cdc6 expression in PBMCs from tumor patients (test group) and non‐tumor individuals as a control group. Chi‐square test with Fisher's exact test was used to analyze the statistical significance of the difference. *P* < .05 is considered as statistically significant difference.

**Results:**

When compared the cdc6 expression in cells from difference sources, we found that A549 tumor cell line had the strongest expression of cdc6, samples from cord blood showed the least expression level, indicating the detection strategy of RT‐qPCR is reasonable. Using this method, we studied whether cdc6 in Peripheral blood could be detected as a biomarker by examining cdc6 expression from PBMCs of patients with lung cancer. We found 20% of patients with lung cancer were cdc6 positive in PBMCs, whereas only 4.26% was found positive in the control group (*P* = .039, *P* < .05).

**Conclusion:**

Cell division cycle 6 has a potential to be used as a circulating tumor cell biomarker for lung cancer. Further study in clinical application is still broad needed.

## 1 INTRODUCTION

Tumor metastasis is a basic feature and an important sign for malignancy, and also a major cause for death.^1^, ^2^, ^3^, ^4^ Hence, early detection and prevention of tumor metastasis are critical for tumor treatment and prevention. It is an independent factor for evaluating cancer disease to detect the tumor cells from peripheral blood.^5^, ^6^, ^7^, ^8^ Researchers are developing methods for tumor cell enrichment from peripheral blood. A variety of methods, such as immunohistochemistry, flow cytometry, and RT‐PCR, have been used in detecting samples from prostatic cancer, breast cancer, non–small‐cell lung cancer, and colorectal cancer.^9^, ^10^ However, low sensitivity and specificity are the major hurdles because of tumor heterogenicity. Some tumor cells intravasated are dormant in the blood stream instead of metastasis. After arriving at a suitable microenvironment, these dormant tumor cells re‐enter a proliferation status, and then stay,^11^ indicating that only proliferating tumor cells possibly are the source of metastasis. DNA replication is the essential event during the cell proliferation. DNA replication initiation proteins including ORC_1‐6_, cdc6 and MCMs are key factors of DNA replication. DNA duplicates actively in tumor cell, which is a major feature of all tumor cells. Thus, it requires involvement of a huge amount of DNA replication initiation proteins. The expression of DNA replication initiation proteins is related to time phase of cell cycle and is reduced or even absent in the static cell. PBMCs including pluripotent stem cells are also in static phase. Hence, we hypothesize that we can detect tumor cells in the peripheral blood from tumor patients by detecting the gene related with DNA replication initiation complex, which could possibly be used as a wide spectrum tumor cell biomarker to monitor the cell proliferation status in peripheral blood of patients. cdc6 is a key factor of DNA replication license system, DNA replication initiation complex, which plays an important role to ensure DNA replicate only once during the one cell cycle.^12^, ^13^ Several studies have showed that cdc6 has the character of oncogene and also plays an important role in the evaluating tumor grade, screening tumor cell and predicting prognosis by probing it in the tissue or exfoliated cells.^14^, ^15^, ^16^, ^17^ Its expression and detection in peripheral blood of patients with tumor remain unknown. In this study, we studied feasibility of using cdc6 as biomarker for circulating tumor cell by examining the expression level of cdc6 in the PBMCs from health individuals, mononuclear cells from cord blood and a tumor cell line A549 with in situ hybridization, reverse transcript quantity polymerase chain reaction (RT‐qPCR) technique. After the method established, we validate it with samples from non‐operating lung cancer patients.

## 2 MATERIALS AND METHODS

### 2.1 Cell culture

Cells from A549 cells line (ATCC^®^ CCL‐185™) were cultured in McCoy's 5A Medium modified with 10% FBS and 1% penicillin and streptomycin (Thermo Fisher Scientific) and maintained in a humidified incubator (5% CO_2_) at 37°C. The cells were harvested for further experiments when their confluency reached 80%.

### 2.2 RT‐qPCR

Total RNA was extracted with TRIZOL reagent (Thermo Fisher Scientific). First strain cDNA was synthesized with Reverse Transcription System A3500 (Promega). PCR was set up with Platinum^®^ Taq DNA Polymerase High Fidelity kit (Thermo Fisher Scientific). Primers design was completed with software primer premier 5.0 based on the sequence of target gene from NCBI gene bank and synthesized by Sangon Biotech. Real‐time qPCR was set up with the LightCycler^®^ 480 Probe master kit (Roche Life Science). β‐actin was used as internal control. Forward primer (5′CCTGTTCTC CTCGTGTAAAAGC3′), reverse primer (5′GTGTTGCATAGGTTGTCATCG3′) for cdc6 and forward primer (5' ACGTGGACATCCGCAAAGAC 3'), reverse primer (5' CAAGAAAGGGT GTAACGCAACT A 3') for β‐actin were synthesized by Sangon Biotech.

### 2.3 RNA probe preparation

Correct PCR product verified by sanger sequencing was cloned in the vector named PEASY‐T3. Then, the vector was transfected to DH5α cells. Positive colonies were picked up by PCR with SP6 primer and target gene forward primer (5′GCTACTGGATTGCCTTAA3′), T7 primer and target gene reverse primer (5′AGCGGGTGCTGTAGTGAG3′) respectively. And sequence of positive colonies was verified with sanger sequencing. Positive colony qualified was used as template for RNA probe which was labeled with Dig RNA labeling kit (Roche Life Science).

### 2.4 Peripheral blood mononuclear cells (PBMCs) separation

About 2 mL whole blood anticoagulated with EDTA was added in 4 mL hemolytic agent (65 mol/L NH_4_Cl, 10 mmol/L KHCO_3_, 0.13 mmol/L EDTA), stood in ice for 15 minutes, and then centrifuged at 1700 *g* for 15 minutes at 4°C. After discarding supernatant then adding 2 mL hemolytic agent again, and repeating as above steps, the pellet was transferred into a new 1.5‐mL EP tube and washed with PBS buffer twice at 1700 *g* for 8 minutes. The pellet was stored at −80°C for the following test.

### 2.5 Simulation sample

About 50 and 100 cells from A549 cell line were added into two tubes containing 1 mL whole blood from health people, respectively, which were anticoagulated with EDTA and named mix sample 1 and mix sample 2. PBMCs were separated according to the method previous mentioned.

### 2.6 In situ hybridization with cdc6 RNA probe

In situ hybridization with cdc6 RNA probe was complied with the procedure of Enhanced Sensitive ISH Detection Kit I (BOSTER WUHAN). About 1.15 × 10^5^cells were evenly spread on the slide treated with 0.1% DEPC and 10% polylysine, and further fixed with 4% formaldehyde/0.1 mol/L PBS containing 0.1% DEPC for 20 minutes at room temperature. 30% H_2_O_2_:methanol (1:25), pepsin, 20 μL Dig labeling RNA probe and DAB was used to eliminate endogenous peroxidase, expose mRNA, hybridize target gene and detect the signal of hybridization, respectively.

### 2.7 Collection of clinical samples

**Figure 1:**
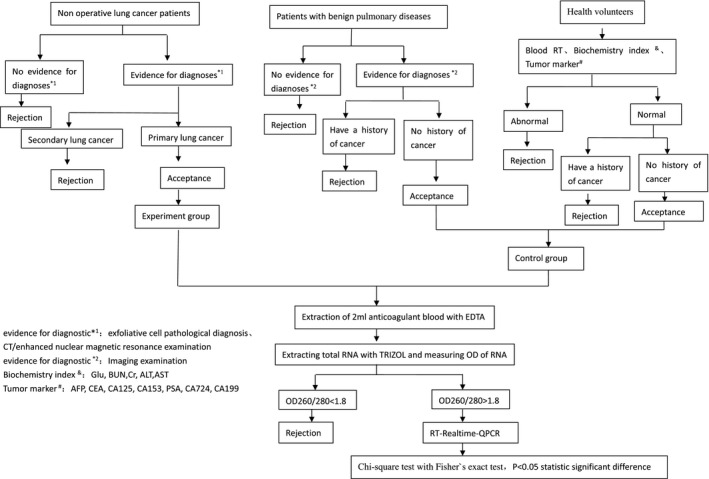
Research design diagram

A total of 63 patients were enrolled in this study, including 40 patients with lung cancer, 23 patients with benign pulmonary disease (Table 1). A total of 24 health volunteers, patients with benign pulmonary diseases, and patients with lung cancer were enrolled as control groups and experiment group, respectively. About 2 mL peripheral blood samples anticoagulated with EDTA were collected from the median cubital vein of these individuals. To avoid tissue cell contamination, the first tube of blood was not used in the study. The inclusion criteria of subjects were shown on the diagram (Figure 1). Approval for the use of human subjects was obtained from the research ethics committee of Guang'anmen Hospital (Beijing, China).

**Table 1:** Study individuals information

	Case	Male	Female	Mean_age_	Min_age_	Max_age_
Lung cancer patients	40	24	16	63	26	85
Benign pulmonary diseases patients	23	14	9	74.26	18	90
Health individual	24	9	15	40.58	26	56

Patients	Lung cancer					Benign pulmonary diseases								
	SCC	Ade.	SCLC	NEC	LC	Pneumonia	Chronic obstructive pulmonary disease	Bronchitis	Acute upper respiratory infection	Interstitial lung disease	Failure of respiration	Bronchiectasis	Bronchial asthma	Pneumo‐coniosis
Case	4	18	9	1	8	8	4	3	2	2	1	1	1	1
Num. of metastasis	3	17	9	0	8									
Num. of cdc6 +	0	4	2	0	2	1	0	0	0	0	0	0	0	0

Abbreviations: Ade, adenocarcinoma; LC, lung cancer; NEC, neuroendocrine carcinoma; SCC, squamous cell carcinoma; SCLC, small‐cell lung cancer.

### 2.8 Statistical analysis

Statistical analysis was performed using SPSS17.0 for windows. Chi‐square test with Fisher's exact test was used. *P* < .05 is considered to indicate a statistically significant difference.

## 3 RESULTS

### 3.1 Preparation of cdc6 RNA probe

A DNA fragment of cdc6 gene was cloned into PEASY‐T3 for RNA probe preparing. The cdc6 sequence was from the GenBank, and the PEASY‐T3‐cdc6 clone was verified by sanger sequencing (Figure S1). The segment of cdc6 for RNA probe preparing was cloned at the downstream of T7 promoter on which cdc6 RNA probe was labeled with Dig by in vitro transcription with T7 RNA polymerase.

### 3.2 Validation of cdc6 expression by in situ Hybridization

**Figure 2:**
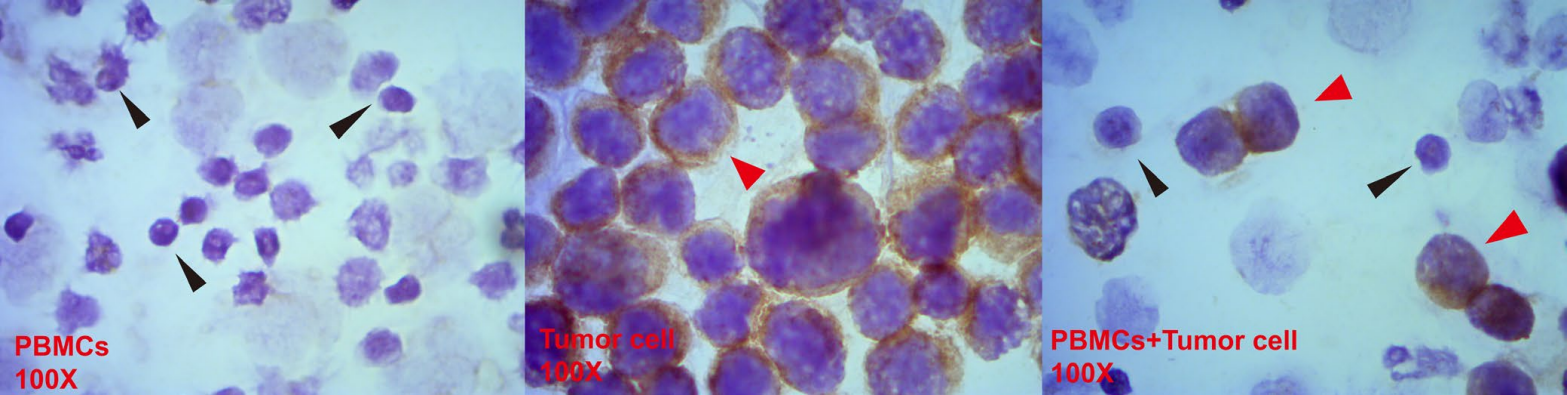
In situ hybridization with cdc6 RNA probe in PBMC and A549 cell. Black arrow and red arrow indicate PBMC and tumor cell, respectively. Brown color shows the signal of in situ hybridization. PBMC (left), A549 tumor cells (middle), and mixture of PBMC and A549 tumor cells (right)

We first asked if our scientific strategy is feasible by examining the cdc6 expression with in situ hybridization technology. Results from in situ hybridization experiments suggest that cdc6 expression in A549 tumor cells was stronger obviously than in peripheral blood mononuclear cells (Figure 2). mRNA of cdc6 was recognized by the probe and showed brown color in the cytoplasm. Cytoplasm of most tumor cells was brown (Figure 2, middle), whereas the cytoplasm of PBMCs from healthy donors was unstained (Figure 2, left). We also mixed A549 cells with PBMCs, and the results clearly showed that PBMCs remain unstained even staining time prolonged from 5 to 45 minutes (data not shown) (Figure 2, right).These results are as expected and suggest that a high specificity of the in situ hybridization method.

### 3.3 Expression level of cdc6 in the tumor cells and PBMCs by RT‐qPCR

After confirming cdc6expression by hybridization, we next asked if cdc6could be detected by a high‐throughput method, qPCR. We used RT‐qPCR to examine the cdc6 expression in A549 cell line, and mononuclear cells from peripheral blood or cord blood of health donors. Results are summarized in Table 2. For cdc6 expression, cells from A549 cell line were the highest, whereas Cord blood sample were the least. To calculate the expression level of cdc6 in cells from difference source, comparative *C*t method was used. β‐actin and PBMCs from health individuals were used as internal reference gene and contrast, respectively. For cdc6 gene expression, any samples from tumor cells, mix samples or PBMCs from cord blood with a 2^−ΔΔ^ *^C^* ^t^>2 or < 0.5 was considered significantly different from health control. We also observed that the expression level of cdc6 increased with the number of proliferation cells. Tumor cell is a typical one. The cdc6 expression in cells from A549 cell line, mix sample1, mix sample2, and PBMC from cord blood was 61.82, 13.93, 25.93, and 0.266 times as much as that in PBMCs from health individuals, respectively. These results further confirm that the cdc6 expression in A549 cell line is higher than that in PBMCs.

**Table 2:** The expression of cdc6 in different cells

	2^−ΔΔ^ *^C^* ^t^
Tumor cell	61.82
Mix sample 1	13.93
Mix sample 2	25.63
PBMC from cord blood	0.266
PBMC	1

### 3.4 Lung cancer patients have high cdc6 expression in PBMCs

Finally, we utilized the established high‐throughput qPCR method to detect samples from patients with lung cancer. The results are summarized in Table 3. We found that 8 out of 40 (20%) patients with lung cancer (PBMC) were cdc6 positive. From 47 individuals of control group, only two were cdc6 positive (4.26%). The difference is statistically significant (*P* = .039, *P* < .05). All of the eigth patients with cancer observed cdc6 positive undergone metastasis. Of all the 8 cdc6‐positive lung cancer samples, six were from small‐cell lung cancer and adenocarcinoma patients, and two were from patients with lung cancer whose tissue classification reports were not available.

**Table 3:** The expression of cdc6 in tumor group and control group

	Positive cases/positive rate (%)	Negative cases	*X* ^2^	*P*
Lung cancer group	8/20	32	5.06	.039
Control group	2/4.26	45		

## 4 DISCUSSION

In this study, we examined cdc6 expression in PBMCs from health subjects and patients with lung cancer. Our results demonstrated a distinct expression profile of cdc6 between these two groups, indicating a potential value of cdc6 as a circulating tumor cell biomarker for patients with lung cancer.

One feature of tumorgenesis is that disordered DNA replication induces aneuploid cells. DNA duplication of tumor cells requires a huge amount of DNA replication initiation proteins. Cdc6 is the known marker of proliferating cells. Its expression is closely connected with time phase of cell cycles and is reduced or even absent in static cells. It has been shown that cdc6 highly expressed in tumor cells than normal cells.^14^, ^15^, ^16^, ^17^ It makes detecting tumor cells in the peripheral blood in patients with tumor possible by detecting cdc6. This could be used as a tool of risk evaluation of tumor relapse or metastasis, and the monitoring of the therapeutic effect.

It is possible to detect the tumor cells in the peripheral blood, lymphonodi and bone marrow at mRNA level by using RT‐PCR technique.^18^, ^19^, ^20^, ^21^ But its clinical application is limited, because negative results can be caused by the absence of some tumor specific genes expression due to the heterogeneity of tumor.^22^, ^23^ To study whether cdc6 could be used as a circulating tumor cell marker with RT‐qPCR technique, we probed the RNA‐seq data from gene browser at Dr Wang zhibin's laboratory of John Hopkins University. Significant difference of the RNA expression between the T lymphocyte from health individuals and HCT116 (data unpublished) was observed, which supports our result that cdc6 expression in tumor cell is higher than that in normal cells. We initially validated the location and expression of cdc6 in different cell types with in situ hybridization and observed that Cdc6 mRNA mainly located in the cytoplasm, and its signal was very clear in tumor cells and also was much stronger than that in normal PBMCs (Figure 2).These indicate a distinct expression pattern between tumor cells and PBMCs, which further confirmed our view of point. In normal condition, stem cells with proliferative potency in peripheral blood are in the state of dormancy.^24^, ^25^ They only re‐enter cell cycle when stimulated by waking up signals. In order to know whether cdc6 can be observed in stem cells, and their influence on this study, we detected the expression of cdc6 mRNA in the PBMC from cord blood and health individuals, cell mixture of PBMCs and tumor cells, and tumor cells with RT‐qPCR. We observed that the expression of cdc6 in both tumor cells and cell mixture are extremely higher than that in PBMCs from cord blood or health individuals, and cdc6 expression in PBMCs from cord blood is lower than that from health individuals (Table 1). This result further supports the opinion that dormant stem cells do not affect the detection of cdc6 in peripheral blood in order to detect tumor cells. This observation is consistent with the finding by Kingsbury et al,^25^ which showed that there is not expression of cdc6 in NIH/3T3 cells in quiescent state (G0) and unlicensed stem cells from colonic mucosa.

To further study whether cdc6 can be used as a biomarker of circulating tumor cells, we detected cdc6 mRNA expression in PBMCs from lung cancer group and two control groups. Although cdc6 was more frequently observed in patients with lung cancer, the sensitivity is much lower than our expectation, which will limit its clinical use. To increase the sensitivity, some strategies for increasing the chance of detecting tumor cells in PBMCs can be further considered in future investigation: (a) to collect more blood, (b) to selectively get rid of PBMCs before detecting tumor cell. We also observed that the expression of cdc6 was not correlated with the number of white blood cells (lymphocytes; data not provided), which means that the increase of WBC caused by some drugs does not raise the level of cdc6. Of all the eight cdc6‐positive lung cancer patients who were in metastasis status, six were from small‐cell lung cancer and adenocarcinoma patients, which is in accordance with the consensus that adenocarcinoma and small‐cell lung cancer are apt to disseminate through blood stream, whereas squamous cell carcinoma easily disseminate through lymphatic system.^26^, ^27^ We also observed two subjects whose cdc6 was positive. One was from health individual, the other was an inpatient with lung infection, whose CT for chest and abdomen showed that lymph nodes of mediastinum and abdomen were enlarged, and whose serum CYFRA‐211 and SCC level were higher than reference value. Cdc6 tests were performed for three times with different PBMCs from this patient, and all of the results were positive. Based on these evidences, the patient should be consider a patient with cancer, but his family members refused to accept further examination because of age factor (87 years old). The other was a young female, and we later found that she was a patient with mild thalassemia. No evidence supported that she was a patient with tumor, except several erythrocytoblasts in peripheral blood. Theoretically, the two cdc6‐positive subjects should be excluded from the study, and thus, no cdc6‐positive subjects had been observed in control group, which shows the high specificity of the study. Further study will be focusing on how to increase the sensitivity of the methodology, and whether it can be used as a new way for risk evaluation of tumor relapse or metastasis, and the monitoring of the therapeutic effect with enlarged cases.

## Supporting information

Figure S1
