# Supplementary figures and images for: A pilot study of cdc6 as a biomarker for circulating tumor cells in patients with lung cancer
Source: J Clin Lab Anal. 2020 Apr 6;34(6):e23245. doi: 10.1002/jcla.23245 (PMC7307357; doi:10.1002/jcla.23245)

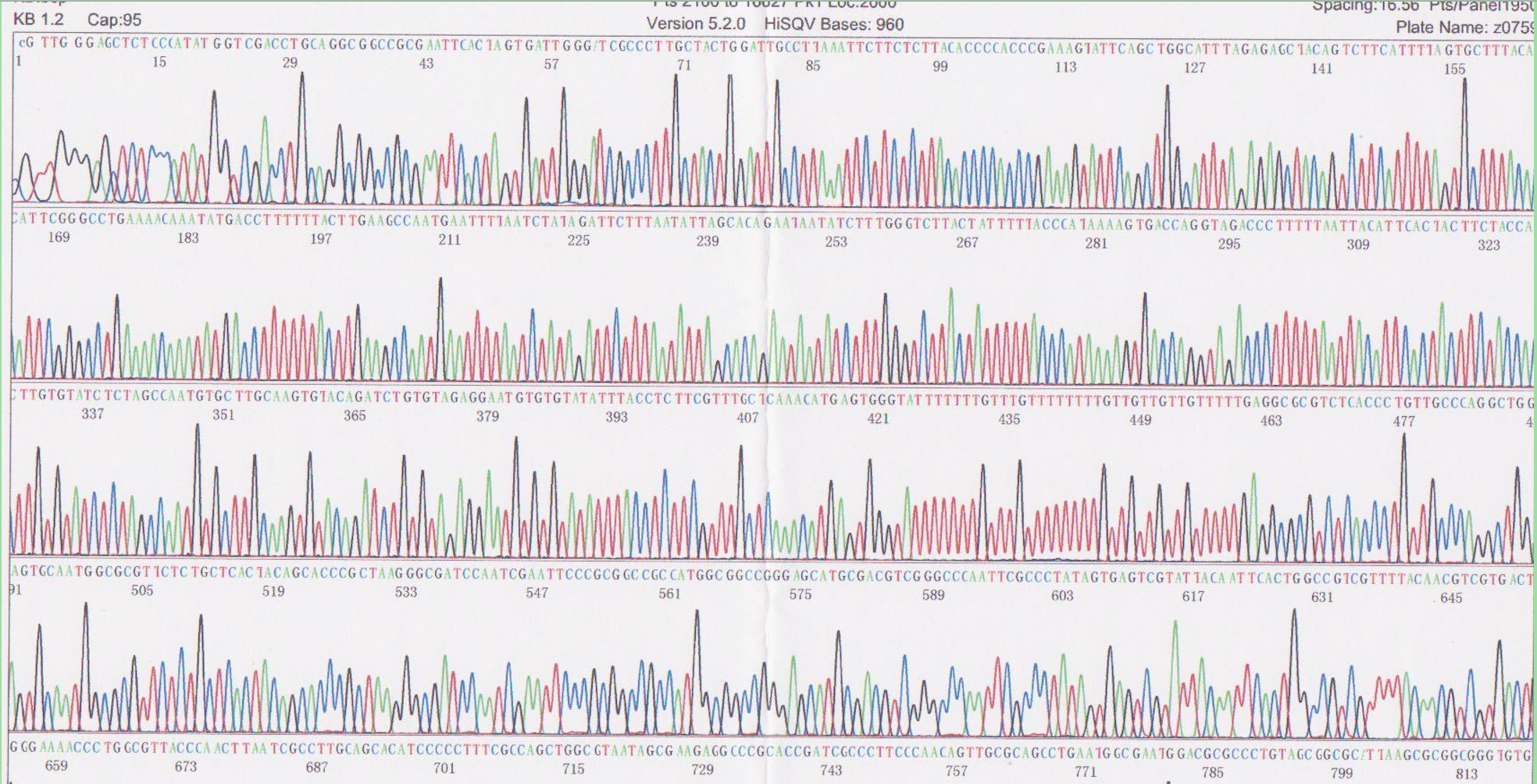

Supplement: Supplementary file 1 — Figure S1 [file JCLA-34-e23245-s001.docx]
